# Actin polymerization state regulates osteogenic differentiation in human adipose-derived stem cells

**DOI:** 10.1186/s11658-021-00259-8

**Published:** 2021-04-15

**Authors:** Bing Sun, Rongmei Qu, Tingyu Fan, Yuchao Yang, Xin Jiang, Asmat Ullah Khan, Zhitao Zhou, Jingliao Zhang, Kuanhai Wei, Jun Ouyang, Jingxing Dai

**Affiliations:** 1 Guangdong Provincial Key Laboratory of Medical Biomechanics and Department of Anatomy, School of Basic Medical Science, Southern Medical University, Guangzhou, China; 2 Central Laboratory, Southern Medical University, Guangzhou, China; 3 Department of Foot and Ankle Surgery, Henan Luoyang Orthopedic Hospital, Zhengzhou, China; 4 Division of Orthopaedics and Traumatology, Department of Orthopaedics, Guangdong Provincial Key Laboratory of Bone and Cartilage Regeneration Medicine, Nanfang Hospital, Southern Medical University, Guangzhou, China

**Keywords:** Human adipose-derived stem cells, Actin polymerization, Osteogenic differentiation, Jasplakinolide, Cell proliferation and migration

## Abstract

**Background:**

Actin is an essential cellular protein that assembles into microfilaments and regulates numerous processes such as cell migration, maintenance of cell shape, and material transport.

**Methods:**

In this study, we explored the effect of actin polymerization state on the osteogenic differentiation of human adipose-derived stem cells (hASCs). The hASCs were treated for 7 days with different concentrations (0, 1, 5, 10, 20, and 50 nM) of jasplakinolide (JAS), a reagent that directly polymerizes F-actin. The effects of the actin polymerization state on cell proliferation, apoptosis, migration, and the maturity of focal adhesion-related proteins were assessed. In addition, western blotting and alizarin red staining assays were performed to assess osteogenic differentiation.

**Results:**

Cell proliferation and migration in the JAS (0, 1, 5, 10, and 20 nM) groups were higher than in the control group and the JAS (50 nM) group. The FAK, vinculin, paxillin, and talin protein expression levels were highest in the JAS (20 nM) group, while zyxin expression was highest in the JAS (50 nM) group. Western blotting showed that osteogenic differentiation in the JAS (0, 1, 5, 10, 20, and 50 nM) group was enhanced compared with that in the control group, and was strongest in the JAS (50 nM) group.

**Conclusions:**

In summary, our data suggest that the actin polymerization state may promote the osteogenic differentiation of hASCs by regulating the protein expression of focal adhesion-associated proteins in a concentration-dependent manner. Our findings provide valuable information for exploring the mechanism of osteogenic differentiation in hASCs.

**Supplementary Information:**

The online version contains supplementary material available at 10.1186/s11658-021-00259-8.

## Background

Actin, a single polypeptide chain globular protein with a molecular weight of 42 kDa, is the primary component of the cytoskeleton. There are two main forms of actin, the globular actin monomers (G-actin) and the polymeric actin filaments (F-actin). Actin plays a crucial role in many biological systems. The polymerization/depolymerization of actin is closely related to numerous cell activities, including the maintenance of cell morphology [2], cell movement [3], cytoplasmic streaming [3], material transport, apical growth, physical, and chemical signal transduction [4–7]. The dynamic remodeling of F-actin plays a crucial role in many diseases, such as cancer [8, 9] and malaria [10–12], and is additionally involved in the wound healing process, embryonic development, and tissue formation [13, 14].

In recent decades, several substances involved in the regulation of actin polymerization and depolymerization have been investigated. These include phalloidin [15], cytochalasin D [16], and latrunculin A [17, 18], which can alter intracellular actin organization [19]. This group also includes jasplakinolide/jaspamide (JAS) [20–25], which was originally isolated from a marine sponge [20, 25–27]. A major advantage of JAS is that it is membrane permeable [28], which makes it an ideal tool for stabilizing or polymerizing actin filaments in live cells. It induces the nucleation of actin filaments, reduces the rate of dissociation of actin subunits from F-actin filaments, and prevents cofilin from severing F-actin filaments, resulting in filament stabilization [27, 29, 30].

Adipose-derived stem cells (ASCs) are a type of adult mesenchymal stem cell derived from fat tissue. They self-replicate and can differentiate in multiple directions [31–34], including the osteogenic, adipogenic [33, 35, 36], chondrogenic [37], and myogenic [36, 38] routes. ASCs are a good cell source for stem cell research [31] because they are widely available, easy to isolate, proliferate stably in vitro, and have a low decay rate [33, 39, 40]. Numerous studies have demonstrated that the polymerization of the actin cytoskeleton can affect cell migration, proliferation, and differentiation [3, 41]. Recent studies have shown that actin microfilament expression increased during the process of osteogenic differentiation. These microfilaments are orderly, thick, and arranged in a filamentous shape [42, 43]. However, how osteogenic differentiation is affected by the extent of actin filament polymerization remains unclear. Therefore, in our study, human ASCs (hASCs) were treated with different concentrations of JAS (resulting in different degrees of polymerization) to study the influence of actin polymerization state on the osteogenic differentiation of hASCs.

## Materials and methods

### Cell culture and jasplakinolide assay

After obtaining informed consent from all patients and approval from the Southern Medical University (Guangzhou, China) ethics committee, leftover subcutaneous adipose tissue was acquired from patients undergoing orthopedic surgery. Human fat tissue was washed with phosphate-buffered saline (PBS) three times, cut into pieces, and then incubated in 0.15% collagenase type I (Sigma, NY, USA) at 37 °C, 5% CO_2_ for 60 min. An equal volume of growth medium containing 10% FBS (Gibco, NY, USA) and 1% Penicillin–Streptomycin (Gibco, NY, USA) was used to terminate digestion. This mixture was centrifuged for 5 min at 250*g* (room temperature), the supernatant was removed, and the pellet was suspended in growth medium and incubated at 37 °C, 5% CO_2_. After 24 h, the medium was completely replaced with fresh medium, and subsequently changed every other day. The cells were passaged at 80% confluence and used between the 3rd and 5th passage (plating density = 6000 cells/cm^2^). After 24 h, cells were treated daily with a growth medium or osteogenic differentiation medium containing different concentrations of jasplakinolide (JAS), or control medium containing the same volume of DMSO. The osteogenic differentiation medium (OS) consisted of DMEM supplemented with 10% FBS (Gibco, NY, USA), 1% Penicillin–Streptomycin (Gibco, NY, USA), 10 mM β-glycerophosphate (Sigma, NY, USA), 100 nM dexamethasone and 37.5 mg/L ascorbic acid (Sigma, NY, USA).

### Cell Counting Kit-8 (CCK-8) assay

Discarding the medium, cells were incubated with CCK-8 solution (Dojindo Cell Counting Kit-8) according to the manufacturer’s instructions (37 °C, 5% CO_2_ incubator). After 0.5 h, absorbance was measured at 450 nm using a microplate reader (Bio-Rad, USA).

### Immunofluorescence assay

Cells were washed two or three times with PBS, then fixed in 4% paraformaldehyde for 10 min, washed three times with PBS, and permeabilized with 0.1% Triton X-100 for 5 min, and blocked in 2% BSA for 1 h at room temperature. Next, cells were incubated with primary antibodies (YAP, 1:700, Cell Signaling Technology, USA; Ki67, 1:500, Abcam, USA) overnight at 4 °C. The corresponding secondary antibody was then added to samples and incubated at room temperature for 1 h. These antibodies included Cy3 labeled goat anti-rabbit IgG (H + L), Beyotime, China; Alexa Fluor 546 goat anti-mouse, Life Technologies, USA; or Alexa Fluor 488 phalloidin, Thermo Fisher Scientific, USA. After washing three times with PBS, cells were incubated with DAPI. The images were observed and captured using a fluorescence microscope (Carl Zeiss; German). Data analysis was performed using ImageJ 1.52 V (NIH, USA).

### Cell apoptosis

Cells were washed three times with PBS and digested with trypsin (without EDTA). Then cells were recovered by centrifugation (500*g* for 5 min), washed with pre-cooled PBS, and then centrifuged (500*g* for 5 min) again (this process was repeated before proceeding). To measure apoptosis, we used the Annexin V-FITC/Propidium Iodide (PI) Apoptosis Detection Kit (Dalian Meilun Biotechnology, China). We added appropriate volumes of working solution to the cell pellet, resuspended the cells, and reconstituted the cells at a density of 1 × 10^6^ cells/mL. Next, we pipetted 100 µL of cell suspension (1 × 10^5^ cells) into a new tube, added both Annexin V-FITC and PI (5 µL), and incubated at room temperature for 15 min. Lastly, 400 μL of the working solution was added to each tube, and the level of fluorescence was detected by flow cytometry (BD LSR Fortessa X-20, BD, USA). FlowJo 7.6.1 software was used to analyze the data Q2 + Q3.

### Wound healing assay

The cells were plated on a fibronectin-coated 6-well plate. After complete attachment, a vertical scratch was made at the bottom of the well with a 10 μL pipette tip. After washing the cells three times with PBS, a complete medium containing different concentrations of JAS was added to each well. The cells were cultured in an incubator at 37 °C, under 5% CO_2_. We observed cell migration at different time points (0, 6, 12, and 24 h). According to the observations, the results at other time points were consistent with the trend at 24 h, so we have only shown images at 0 h and 24 h here. Images of cells were collected using an inverted microscope (1MT-2-21, Olympus, Japan) and the wound area at 0 and 24 h was quantified with the ImageJ software (ImageJ 1.46r, Wayne Rasband National Institutes of Health, USA).

### Transwell assay

We suspended the cells in growth medium without FBS, added 100 μL of the cell suspension to the upper chamber, and then added medium (20% FBS) with different concentrations of JAS to the lower chamber. After 24 h, cells were fixed in 4% paraformaldehyde for 10 min and stained with 0.1% crystal violet (crystal violet-citric acid staining solution, Soleil, China) for 30 min. Images were obtained with an Olympus microscope (1MT-2-21, Olympus, Japan).

### Alkaline phosphatase activity

Cells were washed three times with PBS and permeabilized with 1% Triton X-100 for 30 min. We followed the instructions of the Alkaline Phosphatase Detection Kit (Nanjing Jiancheng Bioengineering, China). The absorbance of each well was measured at 520 nm using a microplate reader (Bio-Rad, USA). The alkaline phosphatase activity in the sample was calculated from the OD value and the protein concentration of the sample.

### Alkaline phosphatase staining

Cells were fixed in 4% paraformaldehyde for 10 min, washed three times with PBS, and incubated with ALP staining solution (Alkaline phosphatase staining kit, Beyotime, China) for 30 min, and then washed three times with PBS. An Olympus microscope was used to acquire images.

### Alizarin red staining

The cells were fixed in 4% paraformaldehyde for 10 min, washed three times with PBS, and incubated with Alizarin red staining solution at room temperature for 3 min. Cells were then washed three times with PBS. An Olympus microscope was used to acquire images.

### Western blotting

The total protein samples were extracted according to the kit instructions (Whole Protein Extraction Kit, Beyotime, China). The protein was separated by SDS-PAGE (voltage of 80/120 V) and transferred to PVDF membranes at a voltage of 60 V for 3 h. The membranes were blocked with 5% nonfat dry milk for 1 h at room temperature, incubated with primary antibody: GAPDH (1:8000, Cell Signaling Technology, USA); β-Actin (1:1000, Bioworld, China); OPN (1:1000, Abcam, USA); RUNX2 (1:1000, Abcam, USA); FAK (1:500, Cell Signaling Technology, USA); Talin (1:1000, Millipore, USA); Vinculin (1:800, Sigma-Aldrich, USA); Paxillin (1:1000, Becton Dickinson and Company, USA); Zyxin (1:700, Affinity, USA); YAP (1:700, Cell Signaling Technology, USA); P-YAP (1:1000, Cell Signaling Technology, USA) overnight at 4 °C with gentle shaking, and incubated with appropriate secondary antibodies: horseradish peroxidase-labeled goat anti-rabbit IgG (H + L) (1:5000, Fdbio Science, China); horseradish peroxidase-labeled goat anti-rabbit IgG (H + L) (1:5000, Fdbio science, China) at room temperature for 1 h. Protein expression was visualized using an exposure instrument (Tanon 5500, Tanon, China). Quantification of western blot data was performed by Gel-pro software.

### Statistical analysis

All experiments were performed in triplicate. Statistical analyses were performed using GraphPad Prism 7.04 software. Results are expressed as means ± standard deviation (SD). A t-test was used for comparison between the two groups, and ANOVA was used for comparison between multiple groups. P < 0.05 was considered to indicate statistically significant results.

## Results

### Effect of different actin polymerization states on proliferation of hASCs

**Fig. 1:**
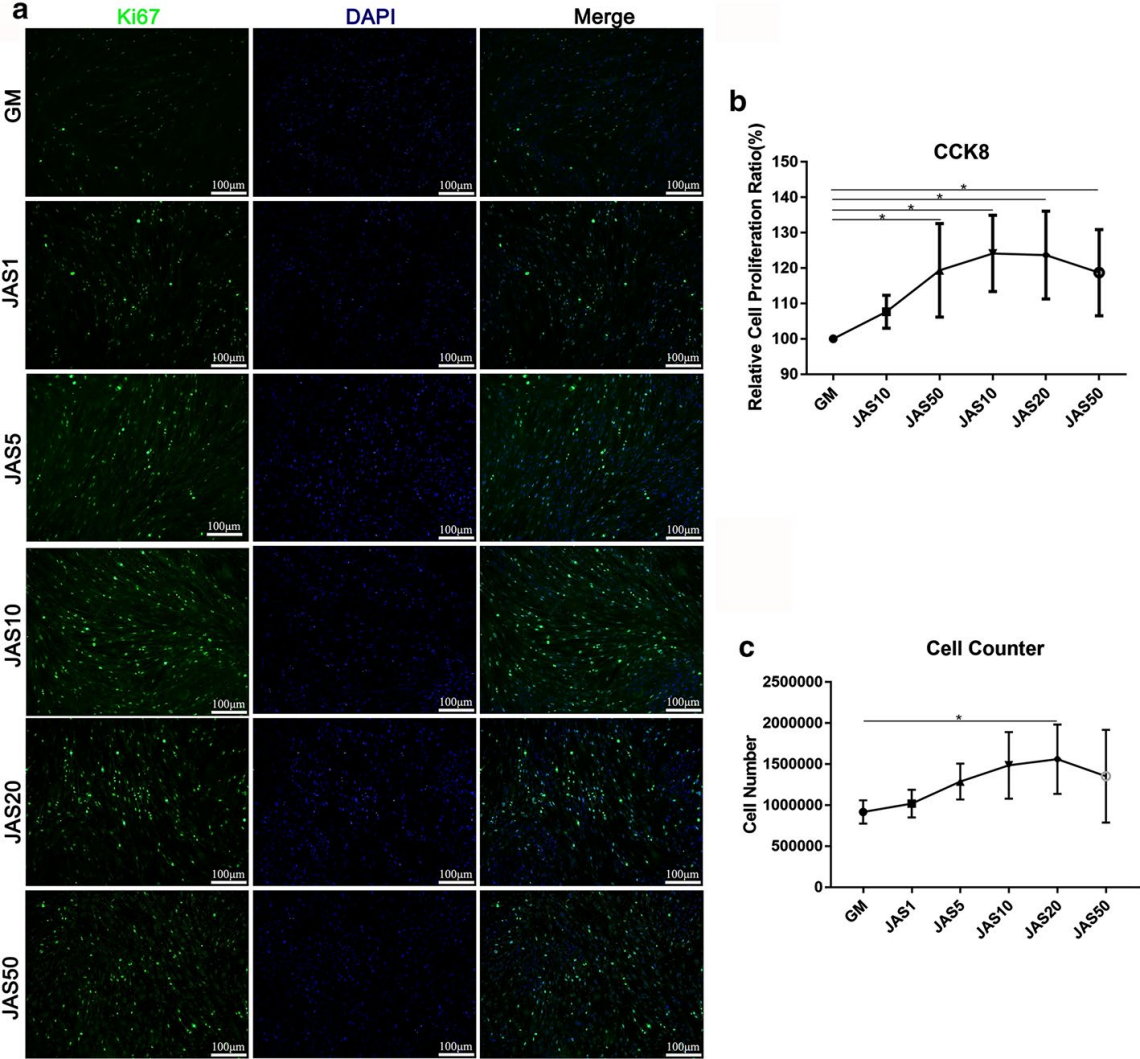
The effect of different actin polymerization states on the proliferation of hASCs. a Control and JAS-treated hASCs stained with Ki67 (green) at day 7. Quantification of the Ki67 positive rate was performed using ImageJ; **b** CCK-8 analysis of hASCs after treatment with JAS for 7 days. Absorbance was measured at 540 nm; (C) Cell number was obtained using a blood cell count plate. Results are expressed as mean ± SD; *p < 0.05. Scale bar = 100 µm

We evaluated the effects of different polymerization states of actin on the proliferation of hASCs. Here, we performed both Ki67 immunofluorescence staining and CCK-8 experiments. The results revealed that the cell proliferation rate in the JAS (1, 5, 10, 20, and 50 nM) groups was higher than that in the control; furthermore, it gradually increased with the increasing JAS concentrations, but significantly decreased in the JAS (50 nM) group (Fig. 1a). The CCK-8 result and cell counter assay were consistent with Ki67 results (Fig. 1b, c). These findings suggest that the actin polymerization caused by JAS at the lower concentration range (1–20 nM) promoted cell proliferation, while the high concentrations JAS (50 nM) inhibited it.

### hASC apoptosis and its relationship with actin polymerization state

**Fig. 2:**
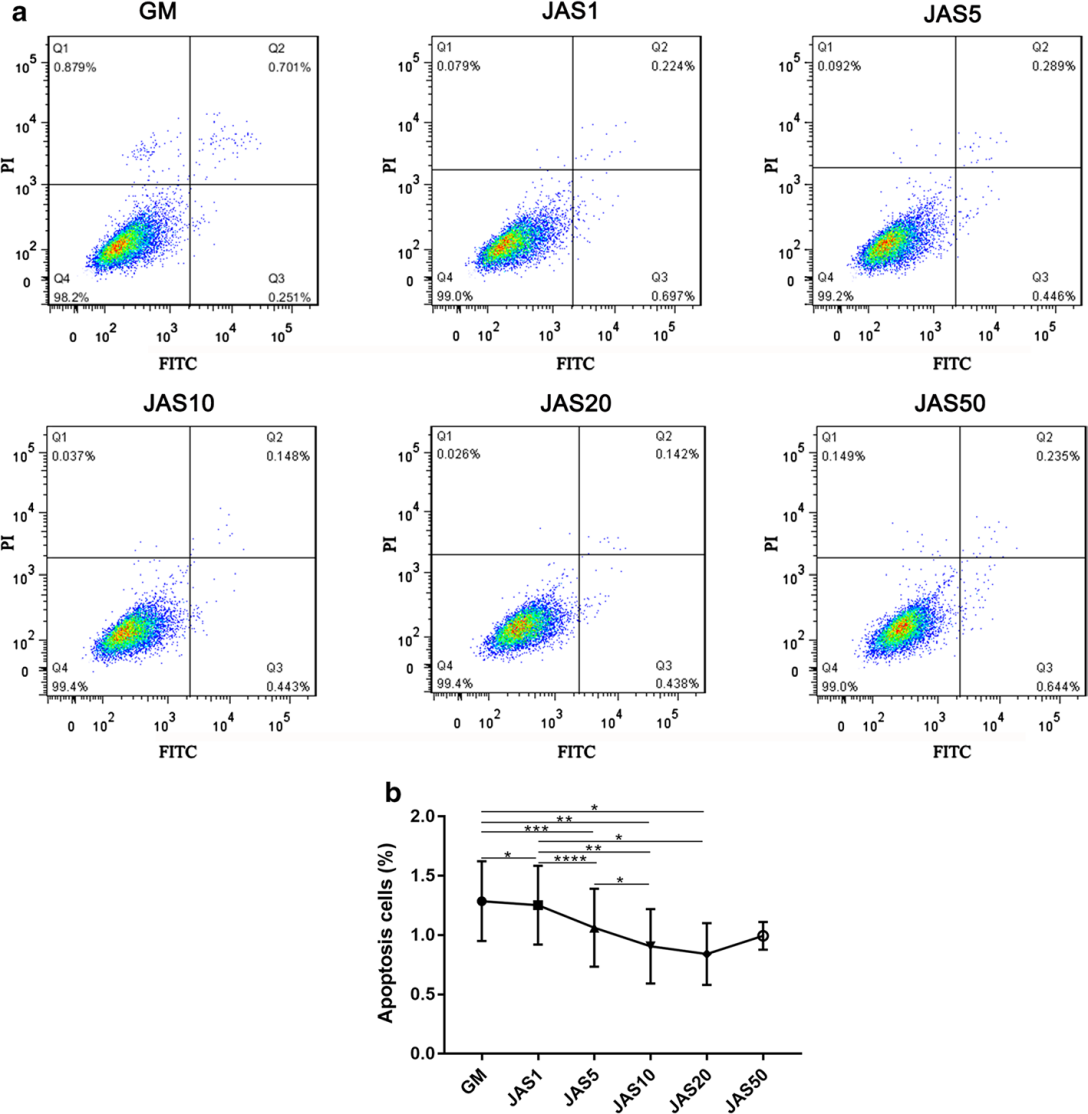
Flow cytometry analysis of the effects of different actin polymerization states on hASC apoptosis. Cell apoptosis was examined by Annexin V-FITC/PI staining. hASCs were treated with JAS for 7 days **a**, and the expression of fluorescence was detected using flow cytometry and analyzed with FlowJo **b**

To investigate the effects of different actin polymerization states on cell apoptosis, we performed the Annexin V-FITC/PI apoptosis assay. Flow cytometry results confirmed that the cell apoptosis rates of the JAS (1, 5, 10, and 20 nM) groups were lower than that of the control group, indicating that a high concentration of JAS promoted cell apoptosis. In contrast, the cell apoptosis rate in the JAS 50 nM group was markedly increased (Q2 + Q3 = 0.879%) (Fig. 2a), indicating that a high concentration of JAS promoted cell apoptosis.

### The influence of different actin aggregation states on hASC migration

**Fig. 3:**
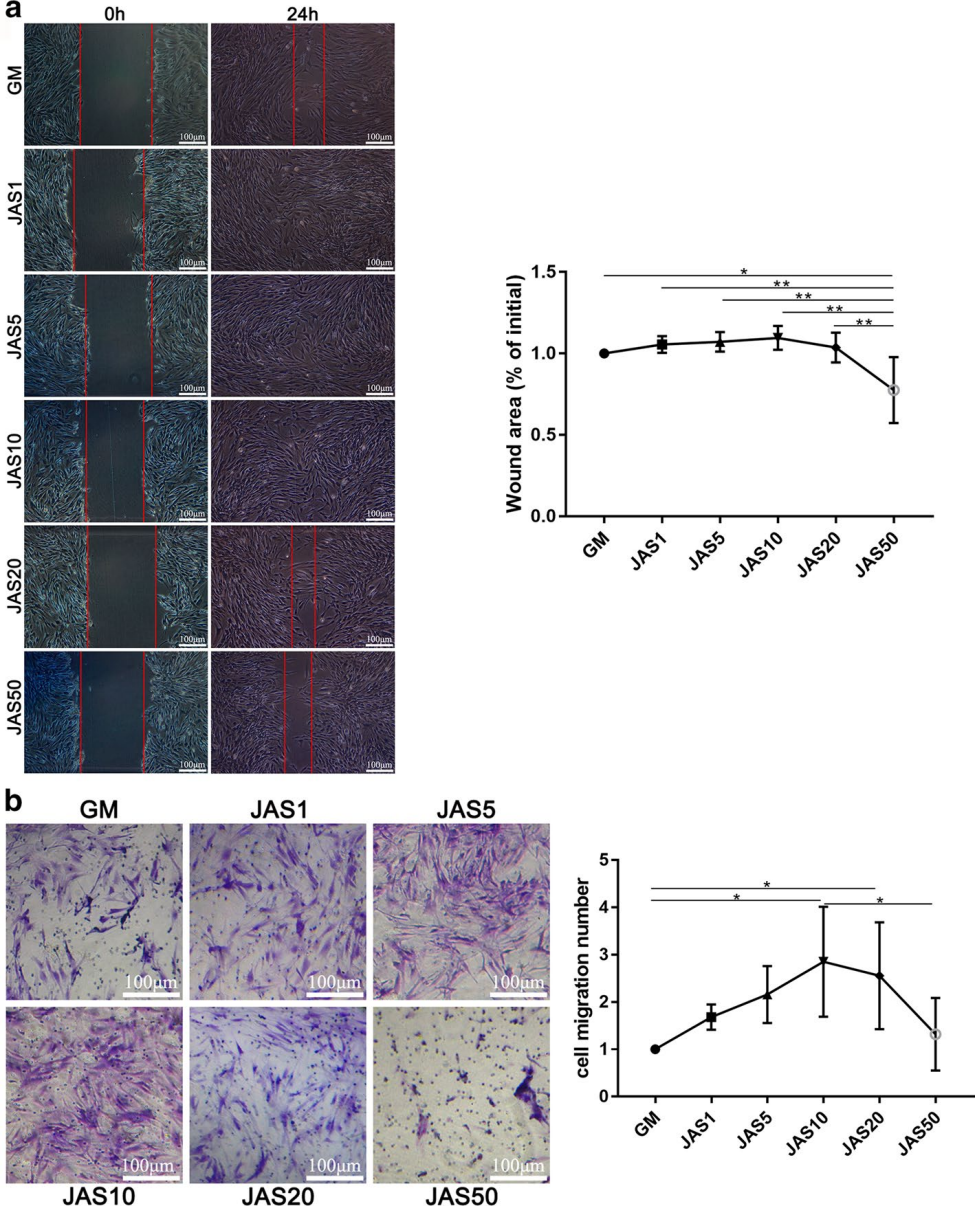
The influence of different actin aggregation states on hASCs migration. **a** hASCs were plated on a fibronectin-coated 6-well plate. When cells were completely attached to the plate, confluent monolayers were scratched, and images were captured by microscopy at 0 and 24 h after the scratch. Quantification of the wound area at 0 and 24 h was performed using Image J. The wound area was calculated as the percentage of the initial wound area (0 h). **b** We suspended the cells in complete medium without FBS, added 100 μL of the cell suspension to the Transwell chamber, and then added medium (20% FBS) with different concentrations of JAS to the lower chamber. After 24 h, cells were stained with 0.1% crystal violet, and cell counting was performed using Image J. Results are expressed as mean ± SD; *p < 0.05. Scale bar = 100 µm

Previous research has demonstrated that the state of actin filaments can regulate cell movement. To study the effect of different polymerization states of actin on cell migration, we conducted wound healing and transwell experiments, respectively. The wound healing results suggested that the healing speed in the JAS (1, 5, 10, and 20 nM) groups was faster than that of the control group, while the JAS (50 nM) group was slower than the control group (Fig. 3a). Transwell results (Fig. 3b) showed that in the JAS (1, 5, 10, and 20 nM) groups, the number of cells in the lower chamber gradually increased but decreased sharply in the JAS (50 nM) group (which was significantly lower than the control group). Transwell results were generally consistent with the wound healing results. These data demonstrated that actin polymerization induced by a low concentration of JAS is beneficial for increasing cell migration. In contrast, a high concentration of JAS induced actin polymerization and negatively regulated cell migration.

### Different actin polymerization states and the maturity of focal adhesions

**Fig. 4:**
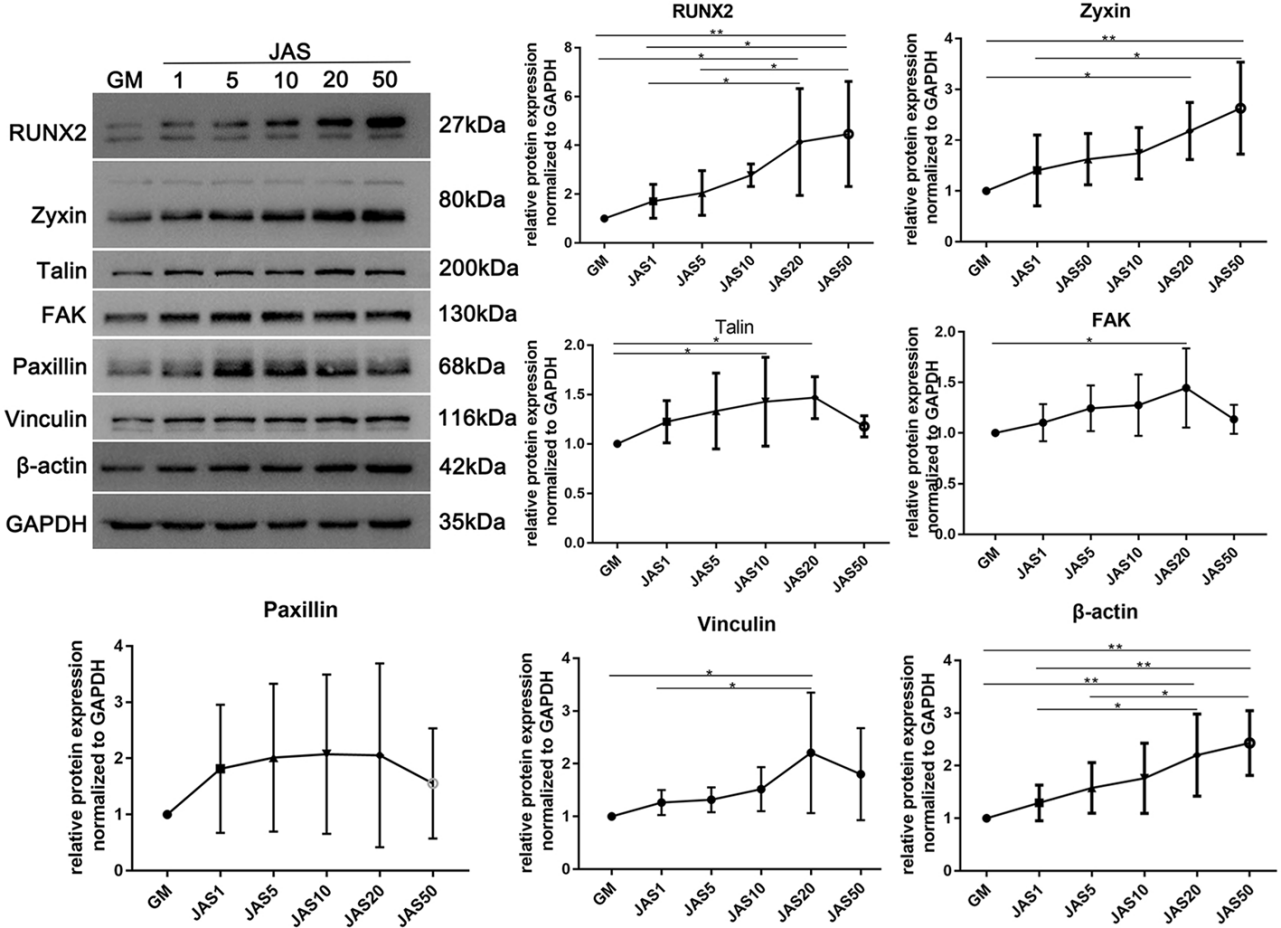
Different actin polymerization states and the maturity of focal adhesions. Western blot analysis was performed to assess the changes in focal adhesion-related proteins of hASCs cultured in medium containing different concentrations of JAS. Samples were collected after 1 week in culture. Quantification of western blot data was performed by Gel-pro software. The relative expression of these proteins was normalized to that of GAPDH. Results are expressed as mean ± SD; *p < 0.05

Studies have shown that the dynamic changes of integrins and focal adhesions are involved in cell migration. Therefore, we hypothesize that the different polymerization states of actin result in differences in cell migration because actin polymerization affects the maturity of focal adhesions. To confirm this, we used western blot analysis to detect the protein expression level of focal adhesion-related proteins (FAK, vinculin, talin, paxillin, and zyxin). The results showed that β-actin, FAK, vinculin, talin, and paxillin were all highly expressed in JAS (1, 5, 10, 20 and 50 nM) groups, but they were highest in the JAS (20 nM) group, and then subsequently decreased in the JAS (50 nM) group. However, interestingly, the expression of β-actin and zyxin showed a continuous increase in the JAS (1, 5, 10, 20, and 50 nM) groups, but there was no decrease in the JAS (50 nM) group (Fig. 4a, b, Additional file 1: Figure S1). Therefore, we believe that actin polymerization could influence the maturity of focal adhesions and that there may be a difference between the influence of low and high actin polymerization states.

### Actin polymerization can promote localization of YAP protein to the cytoplasm

**Fig. 5:**
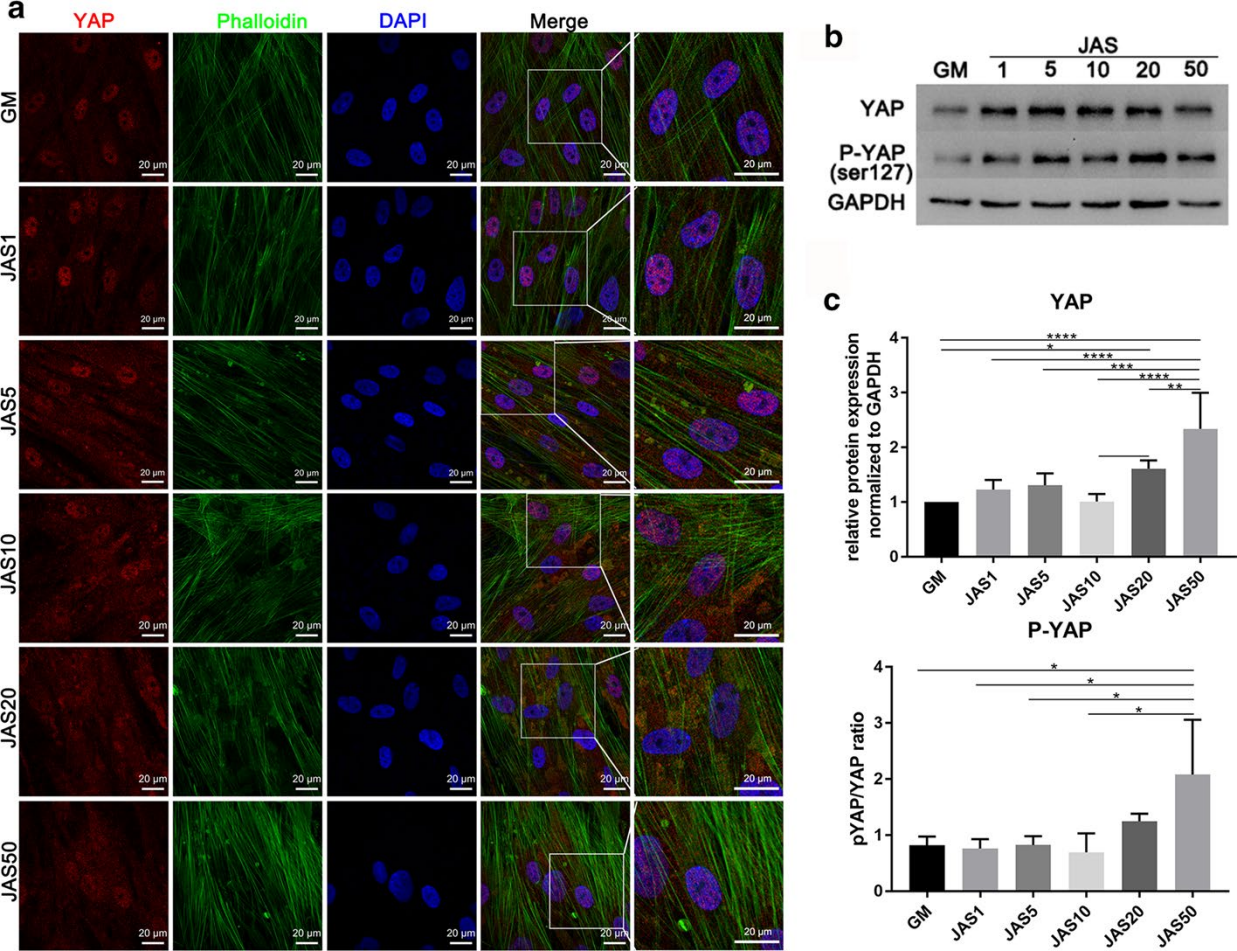
Actin polymerization can promote localization of YAP protein to the cytoplasm. **a** Cells were cultured in medium containing different concentrations of JAS. Then, cells were fixed and immunofluorescence stained with anti-YAP antibodies. Fluorescence for YAP and actin is shown in red and green, respectively. Nuclear staining is represented in blue. All images were obtained using a 63 × oil immersion lens on the confocal microscope; **b** Western blot analysis for YAP and p-YAP (Ser127) of hASCs cultured in medium containing different concentrations of JAS. Scale bar = 20 µm

According to the literature, actin depolymerization can induce the nuclear localization of YAP and regulate gene expression. Therefore, we speculated that different polymerization states of actin might have different effects on the nuclear localization of the YAP protein. To verify this, we used immunofluorescence staining to detect the changes in YAP localization. The results revealed that YAP was mainly concentrated in the nucleus in the control group, but that it began to diffuse into the cytoplasm after cells were treated with JAS. The higher the concentration of JAS, the more apparent was the YAP cytoplasmic localization (Fig. 5a). We also verified this at the protein expression level. The expression of YAP and p-YAP (Ser127) in the JAS (1, 5, 10, 20, and 50 nM) groups was higher than that in the control group (Fig. 5b, c). As the concentration of JAS increased, the ratio of p-YAP to YAP also increased. These data demonstrated that actin polymerization might potently activate YAP by inducing phosphorylation and cytoplasm localization.

### Effects of different actin polymerization states on ALP activity in hASCs

**Fig. 6:**
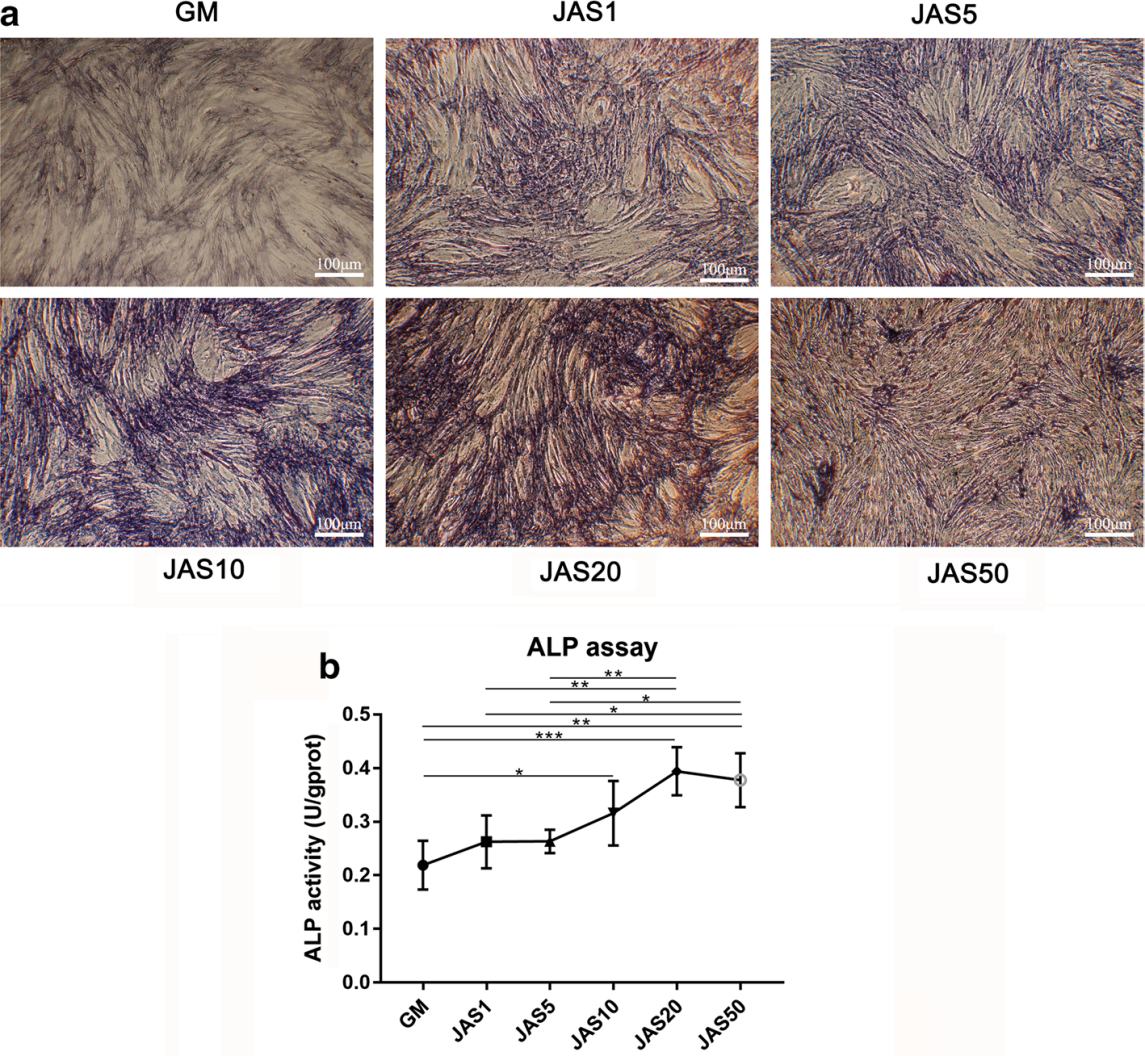
Effects of different actin polymerization states on ALP activity in hASCs.** a** ALP staining of hASCs after treatment with JAS for 7 days; **b** Analysis of ALP activity of hASCs after treatment with JAS for 7 days. Values are mean ± SD; *p < 0.05. Scale bar = 100 µm

Actin polymerization and depolymerization are involved in the regulation of cell differentiation and other biological processes. We believe that JAS may guide the differentiation of cells into osteoblasts. ALP staining and ALP activity assays were performed to explore the effect of actin polymerization on ALP activity in hASCs. The ALP staining results showed that positive staining gradually increased with increasing concentrations of JAS (Fig. 6b), from 0 to 20 nM; however, at the concentration of 50 nM, ALP activity was inhibited (Fig. 6a). The results of the ALP activity assay were approximately consistent with the results of ALP staining. Therefore, our results demonstrate that actin polymerization leads to an increase in ALP activity and content, which can influence cell fate and subsequently direct cells towards an osteogenic lineage.

### Actin polymerization state and osteogenic differentiation of hASCs

**Fig. 7:**
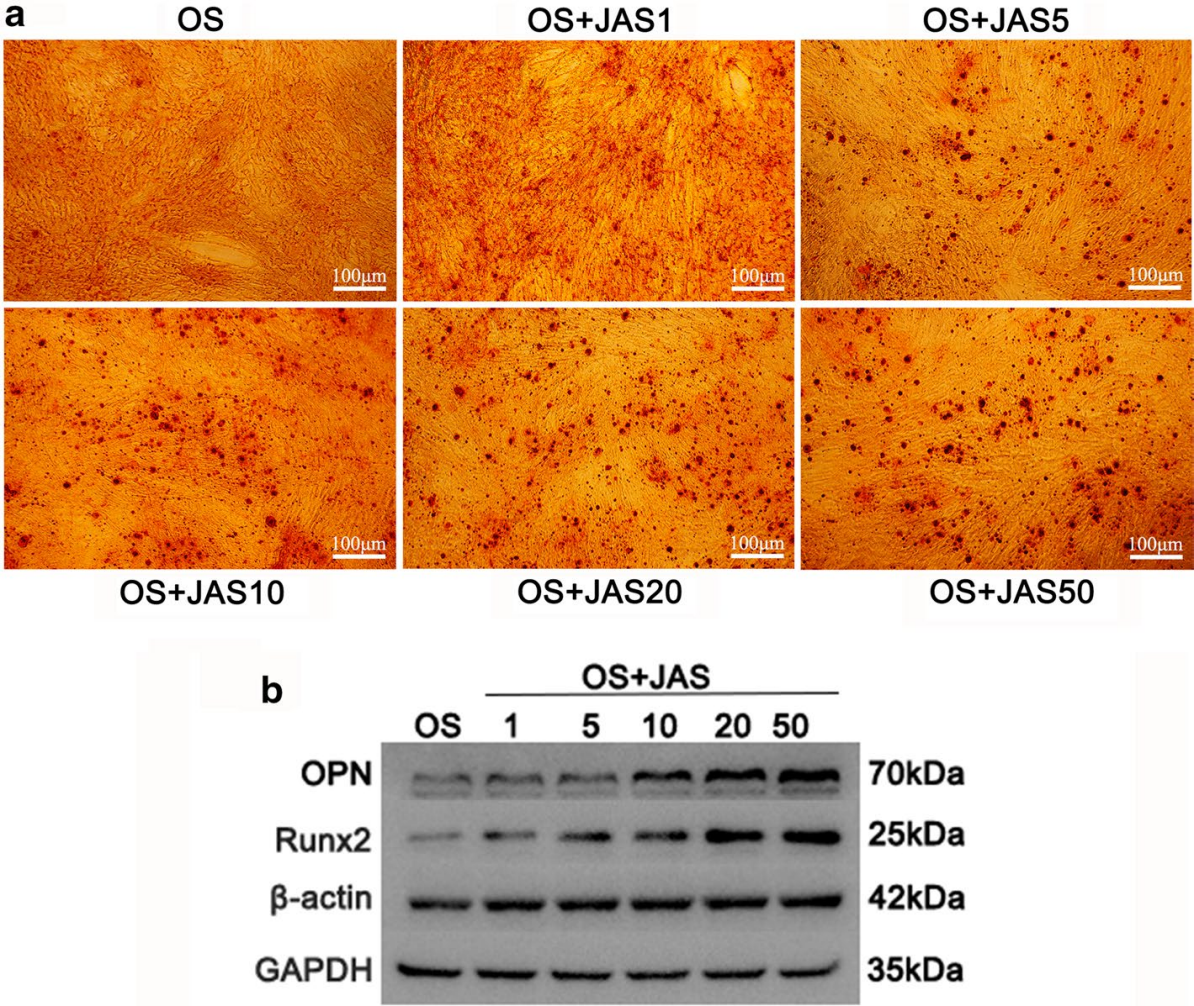
Actin polymerization state and osteogenic differentiation of hASCs. **a** Cells were treated with osteogenic induction medium (containing different concentrations of JAS). They were then stained with alizarin red. **b** Western blot analysis for osteogenic markers of hASCs cultured in the osteogenic induction medium (containing different concentrations of JAS). Samples were collected after one week in culture. Quantification of western blot data was performed by Gel-pro software. The relative expression of these proteins was normalized to that of GAPDH. Results are expressed as mean ± SD; *p < 0.05. Scale bar = 100 µm

The influence of different actin polymerization states on the osteogenic ability of hASCs was investigated by treating the cells with osteogenic induction medium (OS) containing different concentrations of JAS. The alizarin red assay showed that the positive staining in the JAS (1, 5, 10, 20 and 50 nM) groups was higher than that in the control group and strongest in the JAS (50 nM) group (Fig. 7a). Western blotting results demonstrated that osteogenic differentiation markers, such as OPN and RUNX2, were highly expressed in the JAS (1, 5, 10, 20, and 50 nM) groups compared with levels in the control group (Fig. 7b). These results suggest that JAS may stimulate the osteogenic differentiation of hASCs in a concentration-dependent manner.

## Discussion

In this study, we used different concentrations of JAS (an actin polymerization agent) to determine the effects of varying actin polymerization states on the osteogenic differentiation of hASCs. These results, including CCK-8, Ki67 immunofluorescence staining, cell apoptosis, wound healing, and the transwell assay, showed that JAS could promote cell proliferation and migration within a specific concentration range. These data indicated that in the JAS (50 nM) group, cell survival rate, cell proliferation, and migration significantly decreased, while the osteogenic differentiation ability increased. Therefore, we concluded that the polymerization of actin might trigger some osteogenesis-related signaling pathways. These results revealed that the low states of actin polymerization promoted cell proliferation and that high states induced cell apoptosis. This may be due, in part, to JAS cytotoxicity at high concentrations, and the subsequent triggering of downstream signaling pathways related to cell apoptosis.

Cell migration depends on the activity of integrins and the maturation of focal adhesions. According to these findings, the different actin polymerization states resulted in varying focal adhesion maturity (Fig. 4a, b). It is noteworthy that the expression level of zyxin did not decrease at JAS (50 nM), but continued to increase. This trend is consistent with the osteogenic differentiation ability of cells in the OS + JAS (50 nM) group (Fig. 7a, b). Therefore, zyxin, a focal adhesion protein, may have a key role for F-actin to regulate the osteogenic differentiation of cells. In the follow-up research project, we will focus on this key protein (zyxin). Additionally, under the action of a high concentration of JAS (50 nM), the mechanism of the decrease of partial adhesion-related protein expression is still unclear, and further research is urgently needed.

Osteogenic differentiation of hASCs is a complex process in which multiple genes, proteins, and signaling pathways interact with each other [44–46]. The reorganization of the actin cytoskeleton plays an essential role during stem cell differentiation [42]. Previous work has shown that both the location and polymerization of cellular actin aid in the regulation of differentiation. The formation of actin stress fibers is essential for osteogenesis, while its inhibition stimulates lipogenesis [42]. A study found that actin polymerization and depolymerization are involved in the regulation of cell differentiation and other biological processes [44, 47]. A large number of studies have shown that there is an inverse correlation between actin polymerization and adipogenesis, while there is a direct correlation between actin polymerization and osteogenesis [47]. This result is consistent with our findings; that is, the higher the actin polymerization state, the stronger the ability of osteogenic differentiation. Actin depolymerization increases the levels of phosphorylated p38 and ERK1/2 during adipogenesis, and also increases the gene expression of PPARγ [48]. A similar finding was also reported in another study, which showed that myoblast differentiation and osteoblast differentiation are regulated by the p38 MAPK and ERK1/2 pathways of actin filament remodeling [49].

Integrins form the cell matrix connection of actin, through which ECM substances such as fibronectin are connected to the actin cytoskeleton [50]. It has been proved that the cytoskeletal changes necessary for osteogenic formation are integrin-dependent [51–53]. The binding of extracellular components to the cytoskeleton is carried out through the cytoplasmic domain of the integrin that forms the adhesion zone. These adhesion sites are composed of liposomes (protein complexes) that allow mechanical coupling [54–57]. The intracellular actin cytoskeleton senses the cell forces through local adhesome, which leads to the activation of multiple mechanically sensitive pathways, including YAP/TAZ and MKL1 [58, 59]. Integrin-mediated adhesion to ECM is an essential step in determining cell fate during differentiation [60–64]. At the same time, studies have shown that the interaction between cytoskeleton proteins, integrins and mechanical forces can affect cells to change shape, proliferate and even differentiate. It has been found that integrin α5, which is up-regulated during osteogenic differentiation, is an important regulator of osteogenic differentiation. Silencing of integrin α5 eliminated osteogenic differentiation [60].

We know that the actin cytoskeleton is a key determinant of cell shape, which can be explained as the assembly and disassembly of actin filaments [65–67]. Various biological processes such as proliferation and differentiation are affected by cell shape [68–70]. In addition, the actin cytoskeleton-mediated cell morphology changes have been shown to be important for the regulation of MSC lineage commitment and are related to the RhoA pathway [19]. Similarly, the ability of cell shape to determine MSC differentiation is reliant on the actin cytoskeleton and the microtubule skeleton [71]. Therefore, we speculate that the different polymerization states of actin cause changes in cell morphology through the Rho pathway, which may have an impact on the osteogenic differentiation of hASCs. At the same time, the modification of the cytoskeleton caused by Rho GTPase has been found to be the main contributor to the differentiation and migration of mesenchymal stem cells (MSC) [19, 72]. Some studies have shown that these kinases (Rho, ROCK, Rac, LIMK) may be regulators of osteoblast differentiation [73]. These signal transduction pathways can play a role not only by changing the cytoskeleton organization of actin, but also through FAK, JNK and p38 MAPK pathways [44, 74]. The differentiation of osteoblasts is affected not only by cell proliferation, but also by cell geometry [71].

Eunjeong et al. reported that YAP1 binds β-catenin and induces Dkk1, a negative regulator of Wnt signaling, to maintain stemness and prevent osteogenesis [75]. Qiao Y et al. found that YAP promotes the expression of ARHGAP29 to inhibit the RhoA-LIMK-cofilin pathway of apoptosis, destabilizing F-actin, promotes actin depolymerization, and reduces the stiffness of the cytoskeleton [76]. In contrast, Pan and colleagues demonstrated the mechanism by which YAP regulates fat bone formation. They found that YAP interacts with β-catenin to promote osteogenic differentiation and maintain bone homeostasis in the mouse model [77]. A number of studies have demonstrated that alteration of actin dynamics exerts a strong impact on the activity of YAP/TAZ. The various regulatory inputs that determine YAP/TAZ activity are aggregated on the actin cytoskeleton, so induction of filamentous actin (F-actin) bundling by knockdown of F-actin capping or severing proteins promotes nuclear enrichment of YAP/TAZ [78–81]. In contrast, in many cellular environments, treatment of cells with F-actin interfering agents causes retention of YAP/TAZ in the cytoplasm [82, 83]. Since F-actin is known to integrate multiple regulatory signals and participate in various cellular activities, including migration, polarization, and intracellular transmission, Hippo signaling and YAP/TAZ activity may be finely regulated through a complex interlinked regulatory network. Studies have shown that increased actin polymerization can lead to nuclear accumulation of YAP. Our experiments revealed that actin polymerization may effectively activate YAP by inducing phosphorylation and cytoplasmic localization. We speculate that this may be related to different experimental systems: we use different cells, different growth media, and the chemical drugs we use might be trigger other signaling pathways to regulate YAP. Solving these problems still requires further investigation.

In general, we speculate that: (1) the higher the degree of actin polymerization, the easier it is to form a cell shape that is conducive to osteogenic differentiation through the Rho pathway, and at the same time, the thicker the actin fibers are and the more F-actin there is; (2) in the process of JAS promoting actin polymerization to promote the osteogenic differentiation of adipose stem cells, it is likely that integrin regulates the phosphorylation of focal adhesion components zyxin and p38 MAPK, thereby affecting the access of nuclear YAP to promote bone formation.

This study has several limitations. First, we focused on the two-dimensional hASC culture environment, which does not well simulate the in vivo environments. When stem cells are cultured on a two-dimensional plastic/glass surface, the F-actin of the cells is enhanced compared to the cells in the body. Therefore, it will be more interesting to consider the stiffness of the substrate in this study. Secondly, the lack of relevant quantitative experiments is also a shortcoming in this research. In the next step, we will conduct relevant signal pathway research on the subject through experimental methods such as immunofluorescence semi-quantitative analysis, qRT-PCR, gene down-regulation, and gene sequencing, and we will use CytoD as a negative control to further verify this result.

## Conclusion

In summary, in this study, we treated hASCs with different concentrations of JAS to maintain actin in specific polymerization states. We found that increased polymerization of actin promoted osteogenic differentiation, and this may have been achieved by regulating the focal adhesion-related proteins involved in activating YAP. This work forms the basis for further exploration of the mechanisms underlying the osteogenic differentiation of human adipose-derived stem cells.

## Supplementary Information

**Additional file 1: Figure S1**. Different actin polymerization states and the maturity of focal adhesions.

## Data Availability

All the supporting data can be downloaded.

## Abbreviations

JAS: Jasplakinolide
OS: Osteogenic differentiation medium
hASCs: Human adipose derived stem cells
FBS: Fetal bovine serum
CCK-8: Cell Counting Kit-8
ALP: Alkaline phosphatase
