# Supplementary figures and images for: Actin polymerization state regulates osteogenic differentiation in human adipose-derived stem cells
Source: Cell Mol Biol Lett. 2021 Apr 15;26:15. doi: 10.1186/s11658-021-00259-8 (PMC8048231; doi:10.1186/s11658-021-00259-8)

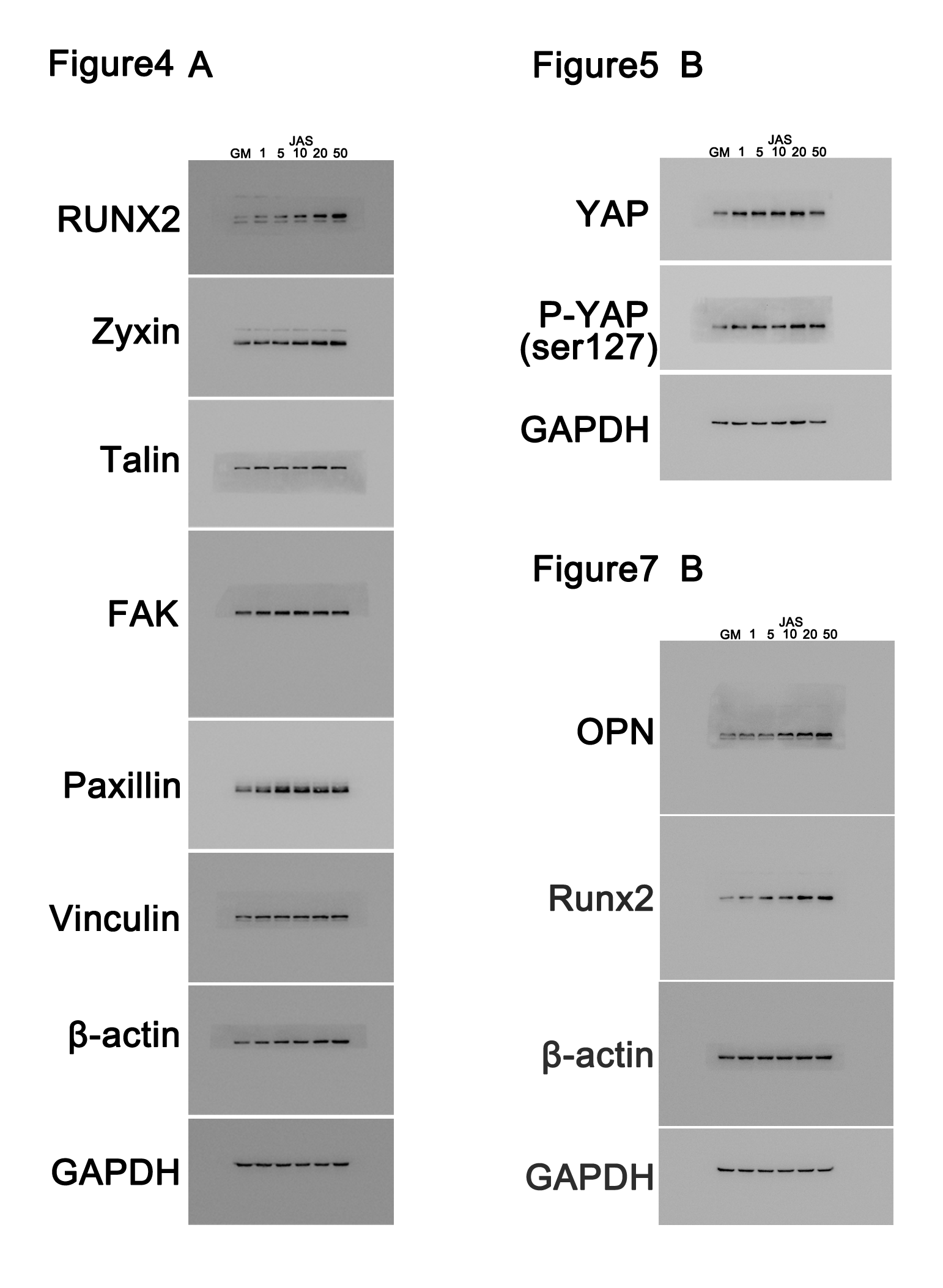

Supplement: Supplementary file 1 — Additional file 1: Figure S1. Different actin polymerization states and the maturity of focal adhesions. [file 11658_2021_259_MOESM1_ESM.tif]
